# Trapeziectomy and ligament reconstruction tendon interposition: an in vivo, patient-controlled, biomechanical study relating to pinch grip

**DOI:** 10.1308/rcsann.2024.0109

**Published:** 2025-04-03

**Authors:** HL Morley, M Argyropoulos, P Souroullas, JAF Read

**Affiliations:** ^1^ West Hertfordshire Teaching Hospitals NHS Trust, UK; ^2^ Hull – York Medical School, UK

**Keywords:** Trapeziectomy, Ligament reconstruction with tendon interposition, LRTI, Pinch grip, Wide-awake local anaesthetic with no tourniquet, WALANT

## Abstract

**Introduction:**

Trapeziectomy and ligament reconstruction tendon interposition (LRTI) is a commonly used procedure in the management of first carpometacarpal osteoarthritis. Debate persists regarding the degree of biomechanical improvement following trapeziectomy and subsequently LRTI. This preliminary, single-centre, in vivo, biomechanical study addresses the biomechanical effect of LRTI following trapeziectomy at the time of surgery, with each participant acting as their own control.

**Methods:**

Trapeziectomy and LRTI were performed under wide-awake local anaesthetic with no tourniquet (WALANT) with a flexor carpi radialis (FCR) donor graft. Key pinch grip was assessed in each participant.

**Results:**

The surgical method that resulted in biomechanically the most significant decrease in grip strength compared with preoperative grip strength was trapeziectomy alone with a mean difference of 4.27kg, which was also statistically significant (Tukey’s test *p*<0.001). Mean post-WALANT key pinch grip strength was 6.4kg (sd 2.61); post LRTI the mean key pinch grip strength was 6.33kg (sd 2.63). Following the surgical procedure (trapeziectomy and LRTI) the mean key pinch grip strength was −0.1kg (95% confidence interval −0.93 to 0.81) (Tukey’s test *p*=1), demonstrating no statistically significant difference between the two values. This indicates that LRTI restores pinch grip strength.

**Conclusions:**

Our preliminary research presents a novel method to evaluate for biomechanical advantages following trapeziectomy and LRTI. This supports the biomechanical advantages of FCR LRTI following trapeziectomy, at the time of surgery. There is scope for this research process to be applied more widely in future.

## Introduction

Our study focuses solely on open trapeziectomy augmented with ligament reconstruction tendon interposition (LRTI) using flexor carpi radialis (FCR) as a donor graft.^1–4^ LRTI using FCR was first described in 1986 using the Burton Pellegrini technique.^2^ The authors used the FCR tendon and a bone tunnel to maintain the trapezial space height and restore the fulcrum at the base of the first metacarpal. This was the technique used in our study.

Debate still endures regarding the advantage of performing LRTI following trapeziectomy. A recent biomechanically focused cadaveric study supports LRTI compared with an unoperated specimen.^5^ By contrast, other studies have concluded that there is no clinical benefit from LRTI with regards to symptoms and patient-reported outcome measures.^2,6^ Notably absent from the current research, however, are in vivo studies scrutinising biomechanical outcomes, such as pinch grip strength at the time of surgery. Our pilot study aimed to determine whether there was a difference in key pinch grip strength, at the time of surgery, following trapeziectomy and subsequent LRTI. This preliminary research also aimed to assess the research method of performing in vivo pinch grip testing under wide-awake local anaesthetic with no tourniquet (WALANT).

The hypothesis of our study was that LRTI following trapeziectomy confers no biomechanical advantage, compared with trapeziectomy alone. This hypothesis was extrapolated from a systematic review that did not report any significant difference.^2^

## Methods

### Research design

We conducted an in vivo, patient-controlled, biomechanical study. Our study design built upon features of anatomical studies (in vitro) that had already been published.^7–9^ Our inclusion and exclusion criteria are detailed in Table 1. All participants were operated upon by a single operator. Blinding the patients was not possible because they needed to follow commands; however, they were unable to see the readings because of a surgical drape. We were unable to blind the operator to the readings. Two individuals agreed readings. Data were collected in vivo, during the operation, prospectively.

**Table 1:** Inclusion and exclusion criteria

Inclusion criteria	Exclusion criteria
• Over the age of 18 • Symptomatic and radiologically confirmed base-of-thumb osteoarthritis • Failure of conservative measures	• Revision trapeziectomy cases • Other planned hand surgery on the same day • Requirement for general anaesthetic • Absent flexor carpi radialis • Inability to consent to surgery

This study was conducted under the Code of Ethics of the World Medical Association (Declaration of Helsinki). Approval was granted by Ethics Committee, REC reference 19/LO/0370.

Adult patients requiring surgical treatment of first carpometacarpal (1^st^ CMCJ) arthritis being listed for trapeziectomy and LRTI, who agreed to having this done under WALANT, were screened for eligibility. Although no participants chose to opt out, they were able do so at any time prior to anonymisation.

### Data collection

Recruitment took place in clinic. On the day of the surgery, patient consent was reconfirmed, and their data were anonymised.

We used an analogue dynamometer (BASELINE^®^ Mechanical Pinch Gauge 30lb/13.6kg) that was in regular clinical use in our hand therapy department. Disposable clear sterile bags were used to ensure sterility while allowing us to take pinch grips readings during the procedure. Each reading was confirmed by the lead operator and independently by the surgical assistant.

### Patient education

The patient was taught how to do a key pinch grip on the dynamometer pre-procedure and their baseline pinch grip readings were recorded. A mean of three readings was recorded. This was conducted with the participant’s arm resting on the operating arm board with the elbow flexed and the shoulder abducted to 90°.

### WALANT

WALANT is a technique that uses local anaesthetic with no tourniquet. Forty millilitres of 1% xylocaine with adrenaline 1:200,000 was diluted to 60ml and infiltrated around the wrist and base of the 1^st^ CMCJ. The quality of the block was confirmed. A key pinch grip reading was taken following the WALANT and a mean of three readings was recorded.

### Trapeziectomy

The trapezium was approached with a Wagner approach. The thenar muscles were elevated to visualise the operative field but without complete removal distally. The thenar muscles were not completely defunctioned. The trapezium was removed piecemeal with bone-nibbling forceps. Confirmation of complete resection of the trapezium was sought before moving on using direct vision. Three key pinch grips were then taken intraoperatively following the completed trapeziectomy and a mean of the three readings was recorded.

### FCR LRTI technique

A stab incision was made proximally over the FCR at 8cm proximal to the wrist crease, the tendon was dissected from the tendon sheath, and the tendon was then split in half and harvested using a Carroll tendon retriever. Following this, the palmer oblique ligament was reconstructed using the split FCR tendon. The split tendon was passed through the first metacarpal and a JuggerKnot^®^ anchor used to secure it to the metacarpal.^10^ The tendon was then looped around itself to create a sling and sutured; following this, the remaining tendon was placed in the trapezial gap.

After performing the LRTI, patients were asked to make another three key pinch grip measurements and a mean of three readings was once again recorded.

The wound was closed using standard surgical techniques.

All data were stored anonymised on a hospital computer. Gender and age of the patients were the only demographic data recorded.

### Statistical analysis

IBM SPSS Statistics^®^ Version 28 was used for statistical analysis. A one-way analysis of variance (ANOVA), along with a post-hoc Tukey multiple comparisons test was used to analyse our results with a *p* value of <0.05 set as statistically significant. The data were evaluated for homogeneity before applying the ANOVA statistical model.

## Results

A total of 15 cases meeting the inclusion criteria were recruited and analysed. None opted out. There were 11 women and 4 men. The mean age was 66.3 years. Thirteen participants were right-hand dominant and two were left-hand dominant. All participants had surgery performed on the right hand apart from one participant in whom the surgery was performed on the left hand; this participant was left-hand dominant. There were no cases of bilateral hand surgery.

The variance among the three methods was assessed for homogeneity and was found to be suitable for application of a one-way ANOVA test (Levene statistic, *p*=0.84). Subsequent performance of one-way ANOVA testing demonstrated a statistically significant mean key pinch grip strength difference in at least one of the intervention groups (*F*=6.39, *p*<0.001). Further Tukey’s post-hoc analysis was performed to determine among which groups the difference was present.

### Key pinch grip strength pre and post WALANT

Mean key pinch grip strength before the WALANT was 9.7kg (sd 3.06). Following the administration of WALANT, the mean key pinch strength was 6.4kg (sd 2.68). The 95% CI of this difference was −4.37 to −2.10.

In terms of percentage change in key pinch grip, following WALANT, there was a 32.97% (2.75kg) statistically significant reduction in mean pinch grip strength (Tukey’s test, *p*=0.04). The percentage reduction in key pinch grip strength ranged from 59.46% to −9.68%, with only a single patient demonstrating an increase.

### Key pinch grip strength following trapeziectomy

Mean key pinch grip strength following WALANT was 6.4kg (sd 2.68). Mean key pinch grip strength following trapeziectomy alone was 4.9kg (sd 2.55). There was a mean reduction of 1.53kg (95% CI −2.32 to −0.74) or 24.4%, ranging from 85.7% decrease to 26.67% increase. There were only three instances in which the pinch grip strength increased (2.94%, 6.25% and 26.67%). The drop among these two groups was not statistically significant (Tukey’s test, *p*=0.44).

### Key pinch grip strength following trapeziectomy and LRTI

Mean key pinch grip following trapeziectomy alone was 4.9kg (sd 2.55). Mean key pinch grip strength following LRTI was 6.3kg (sd 2.63). Following LRTI when compared with trapeziectomy, there was a mean 1.47kg increase (95% CI 0.94 to 2.02) or 44.81%, which was not statistically significant (Tukey’s test *p*=0.47).

### Key pinch grip strength post WALANT compared with post LRTI

Mean post-WALANT key pinch grip strength was 6.4kg (sd 2.61), and post LRTI the mean key pinch grip strength was 6.33kg (sd 2.63). Following the surgical procedure (trapeziectomy and LRTI) the mean key pinch grip strength was −0.1kg (95% CI −0.93 to 0.81) (Tukey’s test *p*=1), demonstrating no statistically significant difference between the two values.

**Figure 1:**
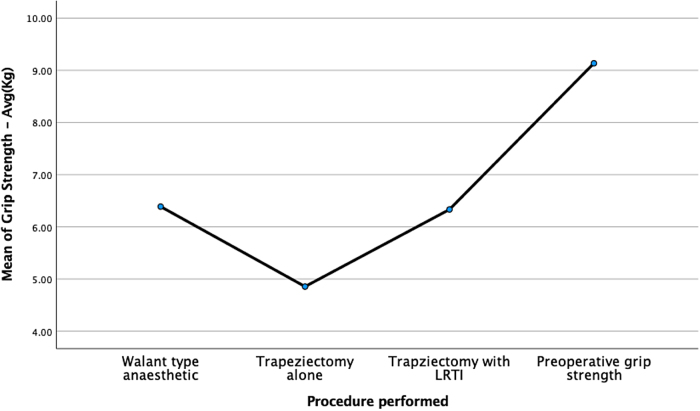
Line graph showing mean pinch grip strength. Further information is given in the Appendix (available online).

Further testing comparing all three interventions with the preoperative grip strength was performed to examine which method or intervention resulted in the most significant reduction in grip strength. Figure 1 and Table 2 detail these figures. The method which resulted in biomechanically the most significant drop in grip strength was trapeziectomy alone, with a mean difference of 4.27kg, which was also statistically significant (Tukey’s test *p*<0.001).

**Table 2:** Tukey’s table

Multiple comparisons					
Dependent Variable: Grip strength – average (kg)					
Tukey’s HSD					
(I) Procedure performed (1 = WALANT, 2 = Trapeziectomy alone, 3 = Trapeziectomy + LRTI, 4 = Preoperative grip)	(J) Procedure performed (1 = WALANT, 2 = Trapeziectomy alone, 3 = Trapeziectomy + LRTI, 4 = Preoperative grip)	Mean Difference (I − J)	Standard error	Significance	95% CIlower bound
WALANT-type anaesthetic	Trapeziectomy alone	1.53	1.02	0.44	−1.16
	Trapeziectomy with LRTI	0.06	1.02	1.00	−2.64
	Preoperative grip strength	−2.75	1.00	0.04	−5.40
Trapeziectomy alone	WALANT-type anaesthetic	−1.53	1.02	0.44	−4.23
	Trapeziectomy with LRTI	−1.48	1.02	0.47	−4.17
	Preoperative grip strength	−4.28	1.00	<0.01	−6.93
Trapeziectomy with LRTI	WALANT-type anaesthetic	−0.06	1.02	1.00	−2.75
	Trapeziectomy alone	1.48	1.02	0.47	−1.22
	Preoperative grip strength	−2.80	1.00	0.04	−5.46
Preoperative grip strength	WALANT-type anaesthetic	2.75	1.00	0.04	0.09
	Trapeziectomy alone	4.28	1.00	<0.01	1.63
	Trapeziectomy with LRTI	2.80	1.00	0.04	0.15

CI = confidence interval; HSD = Honest Significant Difference Test; LRTI = ligament reconstruction tendon interposition; WALANT = wide-awake local anaesthetic with no tourniquet

## Discussion

The data illustrate that trapeziectomy alone is the most biomechanically disadvantageous. Our study demonstrates that LRTI following trapeziectomy does confer a small biomechanical advantage, in vivo, at the time of surgery. When comparing strength between post WALANT (but pre trapeziectomy) and post LRTI, there was no significant difference. Our data indicate that LRTI restores mechanical function to baseline. We hypothesise that this may be explained by a restoration of the fulcrum surrounding the base of the thumb allowing the radius of motion to be restored. This echoes the findings of Koff *et al.*^9^ Therefore, any procedure, of which there are many, that aims to restore the fulcrum at the base of the first metacarpal may produce equivalent results. Other authors have hypothesised that LRTI restores function by maintaining trapezial height.^7,11,12^ However, the significance of trapezial height is controversial. Bhat *et al* demonstrated that no procedure altered the behaviours of the trapezial space left by the removal of the trapezium on radiographic stress views at 1 year. This would indicate that factors other than trapezial height, such as restoring the centre of rotation, are relevant.^11^

There is a trend in our data towards a loss in key pinch grip strength following trapeziectomy, when compared with pinch grip post WALANT. However, this was not true of all cases. Mechanical decoupling of the centre of rotation at the base of the 1^st^ CMCJ could explain this.^9^ Remarkably, in three patients, an increase in strength was noted post trapeziectomy. It is feasible that joint stiffness caused by extensive arthritic changes limited the post-WALANT grip in this group. Thus, the improvement in pinch grip strength post trapeziectomy, when compared with post WALANT alone in these three participants, could be explained by the increased range of movement, which was enabled by removing the joint stiffness caused by the arthritic changes in the joint. Further demographics, including grade of OA, would have been a useful addition to further explain this observed phenomenon in our data.

### Study strengths

The purpose of publishing this preliminary study is in the hope that this method is applied to power a large-scale, multicentre, multiple technique and operator study in future. The need for this has been identified in recent meta-analyses and systematic reviews.^13–17^ An example study design for a larger research project has been provided in the online Appendix.

The central strength of this study is that each participant is acting as their own control. This eliminated the need to record comorbid data and increases the clinical relevance.

Another strength was the concept of having a post-local anaesthetic control. Many cases following WALANT demonstrated a decrease in key pinch grip strength. The exact effect of local anaesthesia on the muscles activating pinch grip function is unclear. The losses could be explained by loss of proprioception and function of flexor pollicis brevis and adductor pollicis muscles. Because all the measurements were taken under the same WALANT they can be reliably compared. A rational explanation for the single patient who demonstrated an increase in key pinch grip strength following WALANT could be the elimination of pain. It is likely that in all participants both effects were present to varying degrees. A useful addition to further research would be evaluating a finger grip in the other digits pre and post WALANT to confirm that the loss of grip strength around the key pinch grip is indeed solely from the WALANT.

Taking key pinch grip readings during the operation is novel and transferable to other locations and procedures. This research method has low costs and low demands on operator time, making it applicable to larger pragmatic trials in future.

### Study limitations

Because this was designed as a preliminary study to evaluate the method, our sample size is small. We were unable to demonstrate a statistically significant improvement in key pinch grip strength with LRTI compared with trapeziectomy alone with this number of participants. An appropriately powered multicentre, multi-operator study would reduce the margin of error. Please refer to our supplementary material for an example.

The study may be criticised because the measurements on the dynamometer were small, and this adds a margin of error at the point of reading. In addition, the key pinch grip strength of many patients with 1^st^ CMCJ arthritis is low.^18^ The decision to use an analogue dynamometer was pragmatic because these are used in routine practice in the UK National Health Service. A digital dynamometer may have provided more precise readings. We did not have funding for this.

We describe lower key pinch grip readings than other authors,^19^ this may be explained by the presence of the WALANT, or indeed the elevation of the thenar muscles required in the Wagner approach during surgery.^18^

Some may question the validity of the results because of the lack of follow up. Our study did not analyse whether this biomechanical advantage was sustained long term. We acknowledge that the lack of longer-term follow up is a major limitation. It is vital to elicit whether LRTI pinch grip strength is short lived or sustained for a longer period because LRTI increases theatre time and introduces a greater risk of complication from the donor site. Indeed, success following an operation depends upon more than intraoperative results; however, we wanted to focus purely on one objective measurable outcome. Although we acknowledge that longevity of surgical outcome, including patient-reported outcome measures, are a crucial part of the debate, this was beyond the scope of this research, which focused on in vivo objective measurements of pinch grip strength at the time of surgery.^20,21^ In addition, we acknowledge that the use of validated patient-reported outcome measures such as QuickDASH alongside final postoperative pinch grip readings could add more meaningful clinical relevance to this study.^22^ Some authors have observed some correlation between pinch grip strength and QuickDASH score; however, this research is not currently reliable enough to use the two outcome measures interchangeably.^23^ Moreover, there has been reported correlation between grip strength ratios and DASH scores.^24^ Grip strength ratio could be used as an alternative outcome measure in future.

There may be a theoretical inaccuracy added by the reducing efficacy of the WALANT during the procedure; the authors note that the average surgical time between these readings was 21min and are confident that this would be minimal, if present at all.

Future changes should include blinding the surgeon and patient to the readings taken and introducing multiple operators in multiple centres. We acknowledge that this is a single-operator study, which could represent a potential source of bias.

## Conclusion

This preliminary research offers a novel, in vivo, biomechanical insight into the direct effects of trapeziectomy and LRTI using an FCR donor. In vivo, at the time of operation, the addition of LRTI using an FCR tendon graft does restore key pinch grip strength. Our novel design meant that each participant functioned as their own control. This research design lends itself to being extended to include other methods, as well as multiple operators, and longer-term biomechanical results. Combining this study design on multiple surgical methods with patient-reported outcome measures would provide more meaningful data to the debate on the management of basal thumb arthritis in future.
